# Knowledge of Arsenic in Drinking-water: Risks and Avoidance in Matlab, Bangladesh

**Published:** 2006-09

**Authors:** Sonia N. Aziz, Kevin J. Boyle, Mahfuzar Rahman

**Affiliations:** ^1^ Virginia Tech, 208-A Hutcheson Hall, Blacksburg, VA 24061, USA; ^2^ ICDDR,B, GPO Box 128, Dhaka 1000, Bangladesh

**Keywords:** Arsenic, Water pollution, Water supply, Drinking-water, Risk factors, Arsenic exposure, Awareness, Bangladesh

## Abstract

Widespread contamination of arsenic in Bangladesh has been jeopardizing the health of millions of people. Residents of Matlab, Bangladesh, are among the millions at risk. Using bivariate models in the analysis of survey data, knowledge of health risks and avoidance of arsenic exposure in response to widespread contamination of arsenic for residents of Matlab were estimated. The models examined individuals' knowledge of an arsenic problem in the household and knowledge of specific illnesses caused by arsenic exposure. The likelihood of avoiding exposure to arsenic contamination was further examined. Results of the estimation showed that individual's knowledge of arsenic problems in the household was gathered through awareness campaigns and by word of mouth and that knowledge of illnesses was predicated on education, health, presence of children, elderly and young women. Adoption of avoidance measures was not affected by exposure to arsenic-information sources, but level of education had a statistically significant positive effect on the decision to avoid arsenic exposure. Lack of convenience of safe drinking-water practices lead people to persist in drinking arsenic-contaminated water.

## INTRODUCTION

The demand for environmental quality in developing countries is generally considered to be relatively low due to poverty. The demand for safe drinking-water, for example, may be overwhelmed by other competing needs for survival. It has been conjectured that lack of awareness about the adverse effects of degraded environmental quality, combined with poverty, keeps the demand for resources such as safe drinking-water relatively low (1). However, if the health risks associated with contaminated water are properly understood, the need for survival may dictate a higher demand for safe drinking-water.

Contaminated drinking-water is a major health hazard in developing countries where infectious diseases caused by pathogens and parasites make up the most common and widespread health risk. The effects of diseases from pathogenic bacteria are immediate and debilitating, even affecting the taste and appearance of drinking-water (2). Despite these obvious ill effects and inexpensive water-purification methods, adoption of safe drinking-water practices is not prevalent. Gadgil showed that many people were unaware of the link between contamination of water and diarrhoea—indicating that awareness of the link between water and disease risk is an important condition for people in developing countries to demand safe drinking-water (3).

Naturally-occurring arsenic in groundwater of South and South-East Asia has also been jeopardizing the health of millions of people who have been drinking contaminated water for years. In areas with natural geological contamination, such as Bangladesh, drinking-water from wells containing high levels of inorganic arsenic can cause serious health consequences, such as skin lesions, cancer, and death (4). Unlike contamination of drinking-water by pathogens, arsenic does not affect the taste or appearance of drinking-water and, moreover, the health effects from ingesting arsenic-contaminated water appear very slowly. For example, the average latency for appearance of skin lesions may be 23 years from the first exposure (5). Use of arsenic-contaminated water may be predicated on lack of awareness of the dangers posed by such action. With taste and appearance not being an issue and with health risks being an abstract notion, it is essential for public-health officials to increase public awareness of risks of arsenic to change the behaviour of people who use this water.

Previous studies attempting to explain and predict health-related behaviours have identified that perceived negative consequence of taking a health action is the most influential variable for predicting actions taken to avoid health risks (6). Other studies have shown that the perceived effectiveness of actions taken to avoid exposure and events that motivate people to take such action (such as public-awareness campaigns) leads to change in behaviour. Results from these studies showed that more knowledge of risks improved the perceived effectiveness of actions taken to avoid exposure. Further, it has been shown that perceived risk may decrease with increase in knowledge (7).

Water sources free of arsenic may be few and far between, taking a practical toll on a person's time available for work. Such perceived negative consequences stemming from the lack of arsenic-free groundwater are complicated by the trade-off of health risks from consuming pathogen-contaminated surface water. Loss of convenience may outweigh the long-term costs associated with obtaining safe drinking-water. This ambivalence or reduced concern may be mitigated by individual household characteristics, such as the presence of children in the household—it is more likely for a child to contract diseases from arsenic exposure within their lifetime that it is for the contraction of such diseases within a parents' lifetime (5). By disseminating knowledge on health risks associated with arsenic exposure, a successful public-awareness campaign could change health behaviours. Accordingly, the demand for safe drinking-water may reflect mitigating factors, such as the presence of children in the household.

To assess people's awareness of arsenic-related health risks, Ahmad *et al*. investigated the extent to which households in Bangladesh are aware of the consequences of consuming arsenic-contaminated water from tubewells (8). Most (87%) respondents were aware of the problem, but few were aware of the serious health risks associated with drinking arsenic-contaminated water. The study also found that radio, television, government and non-governmental agencies were important sources of information of arsenic, followed by members of the family and other residents of the village. Ahmad *et al.* also found that 69% of households in the study area changed their drinking-water source due to contamination with arsenic. While the 2003 study done by Ahmad *et al.* looks at the household's awareness of the arsenic problem and related concerns, no statistical analysis was undertaken to explain households' awareness and households' knowledge of arsenic risks.

The research presented in this paper focuses on a statistical analysis to identify the factors that significantly explain knowledge of arsenic contamination in Matlab, Bangladesh. The specific objectives of this study were to: (i) investigate factors affecting the individuals' knowledge of arsenic contamination in tubewell water in the household, (ii) investigate factors affecting public knowledge of illnesses associated with arsenic exposure in drinking-water, and (iii) investigate household avoidance of exposure to arsenic contamination in drinking-water. A survey was conducted to collect data to address these objectives.

Information from the analyses of these objectives will assist policy-makers in considering the effectiveness of current education efforts and in crafting future public-awareness campaigns of arsenic risks. While results from the study relate to Matlab, Bangladesh, the general findings are of pertinence to other rural areas in the developing world.

## MATERIALS AND METHODS

**Fig. 1.**
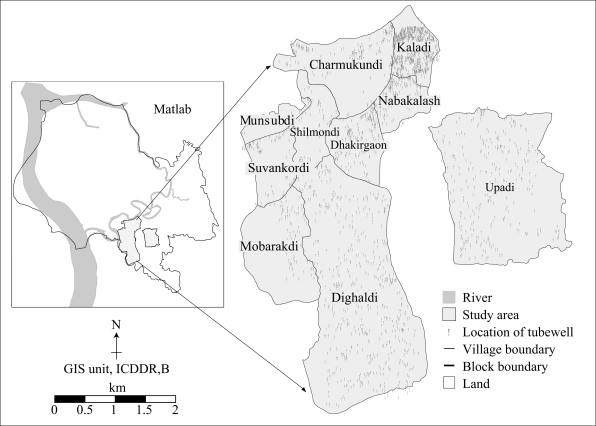
Distribution of tubewells in the study area (Block A) in Matlab, Bangladesh

In-person interviews were conducted in Matlab, Bangladesh, during March-June 2004, under the auspices of International Centre for Diarrhoeal Disease Research, Bangladesh (ICDDR,B). Matlab has seven sub-divisions—A through G—for major ongoing research activities. This study is an addendum to an ongoing project titled “Arsenic in tubewell water and health consequences,” which is a joint effort of ICDDR,B and other collaborating institutions. The study was performed on a stratified random sample of the population in Block A (Fig. 1). The total target sample size for the study was 3,000 households. Enumerators interviewed 2,800 households.

The first section of the survey was designed to collect sociodemographic data. The second section included questions about individuals' awareness of various arsenic-related issues. Section three was designed to reveal household sources of drinking- and cooking-water, including questions on avoidance of exposure to arsenic-contaminated drinking-water. The survey was pre-tested by administering the instrument to 40 people outside the sample area.

Responses from the selected survey items were used for defining binary variables representing individuals' knowledge of arsenic contamination in the household (Objective i), knowledge of arsenic diseases (Objective ii), and avoidance of arsenic exposure (Objective iii). Addressing the first objective involved a variable representing knowledge of an arsenic problem in the household (household arsenic problem). The variable—household arsenic problem—was based on answers to the question of whether the respondent currently or has ever had an arsenic problem in their households.

Addressing the second objective involved a set of variables representing households' knowledge of health consequences of arsenic exposure. The specific health consequences represented by the dependent variables here are knowledge of gangrene (gangrene), knowledge of cancer (cancer), knowledge of skin lesions (skin lesions), and knowledge of death (death) due to long-term arsenic exposure. The answers revealed a wide variation in knowledge of health consequences of arsenic exposure allowing us to examine the link between the knowledge of health consequences of arsenic and the decision to avoid arsenic exposure.

Implementing the third objective involved a variable representing the decision of households to avoid exposure to arsenic-contaminated drinking-water (switch source); this is an indicator for avoidance measures. This dependent variable is based on answers to the question of whether the respondent has ever switched away from using red tubewells that identify elevated arsenic levels.

The investigation of the effects of respondent characteristics on the variables described above was done using a probit model. A probit model is a statistical model in which the dependent variable y*_i_* is either one or zero:

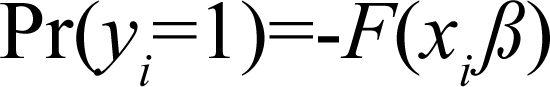


Here Pr(*y_i_*=1) denotes the probability that the dependent variable is 1 (e.g. have an arsenic problem), F is a normally-distributed cumulative distribution function, *x_i_* is a vector of household characteristics, and β is a vector of parameters to be estimated that explains how household characteristics affect each of the six dependent variables (household arsenic problem, gangrene, cancer, skin lesions, death, and switch source). One equation is estimated for each of the six dependent variables defined above.

The independent variables (*x_i_*)—household characteristics that were used for explaining each of the dependent variables—are defined in Table 1. Knowledge of an arsenic problem in the household (household arsenic problem) is specified as a function of children, age, elderly, W13_25, W26_49, education, TW (tubewell) red, arsenic-information sources, concern, poor health, TW distance, and SW (surface water) distance. We conjectured that children, age, presence of young women, presence of elderly, education, arsenic-information sources, concern, and poor health would have a positive effect on knowledge of an arsenic problem in the household (9, 10). The status of tubewell (TW red) was also conjectured to have a positive effect on knowledge of an arsenic problem—the purpose of painting the tubewell red is to raise awareness of arsenic in drinking-water (11). Distance to surface water (SW distance) may have a positive or negative effect on knowledge of a problem. Proximity to surface water may mean that individuals have a lower chance of being exposed to arsenic problems. On the other hand, proximity to surface water may increase the chances of contamination by pathogens, which may prompt the respondent to seek knowledge on water-contamination problems, including contamination with arsenic.

**Table 1.** Variable definitions, expected effects, and means

Variable	Definition	Expected effect on household arsenic problem	Expected effect on knowledge of illness	Expected effect on switch source	Mean
Household arsenic problem	1 if arsenic problem in household, 0 otherwise	NA	+	NA	0.69
Gangrene	1 if the respondent believes that arsenic exposure causes gangrene, 0 otherwise	NA	NA	NA	0.07
Cancer	1 if the respondent believes that arsenic exposure causes cancer, 0 otherwise	NA	NA	NA	0.12
Skin lesions	1 if the respondent believes that arsenic exposure causes skin lesions, 0 otherwise	NA	NA	NA	0.80
Death	1 if the respondent believes that arsenic exposure causes death, 0 otherwise	NA	NA	NA	0.22
Switch source	1 if the respondent switched source from use of tubewell to other sources due to arsenic contamination, 0 otherwise	NA	NA	NA	0.34
Children	1 if children in household, 0 otherwise	+	+	+	0.76
Age	Age of the respondent in years	+	+	+	43
Elderly	1 if persons were aged over 60 years in household, 0 otherwise	+	+	+	0.44
W13_25	1 if women were aged 13–25 years in household, 0 otherwise	+	+	+	0.55
W26_49	1 if women were aged 26–49 years in household, 0 otherwise	+	+	+	0.79
Education	Indexes four levels of schooling:	+	+	+	0.9
	0=None				
	1=Can sign name				
	2=Attended school up to 5^th^ grade				
	3=Attended school up to 10^th^ grade				
	4=Attended school up to 11^th^ grade and above				
TW red	1 if tubewell currently in use has been painted red (red paint signifies arsenic contamination), 0 otherwise	+	NA	+	0.39
Arsenic information sources	Composite additive variable indexing sources from which the household learned about arsenic risks	+	+	+	2.83
	Government=1, 0 otherwise				
	Non-government=1, 0 otherwise				
	Family=1, 0 otherwise				
	Friends=1, 0 otherwise				
	Neighbours=1, 0 otherwise				
	Public notices=1, 0 otherwise				
	Television=1, 0 otherwise				
	Radio=1, 0 otherwise				
Concern	1 if households were concerned about health hazards from arsenic exposure, 0 otherwise	+	NA	+	0.63
Poor health	1 if the respondent was in poor health, 0 otherwise	+	+	+	2.56
TW distance	Distance from home to tubewell currently in use in feet	+	NA	+	86.96
SW distance	Distance from home to surface water currently in use in feet	?	NA	-	67.19

NA=Not applicable;

SW=Surface water;

TW=Tubewell

Knowledge of each illness caused by arsenic exposure was estimated as a separate model. Each of the knowledge of illness variables indicates whether the respondent believed that arsenic causes gangrene, cancer, skin lesions, or death. Each of these variables was specified as a function of household arsenic problem, children, age, elderly, W13_25, W26_49, education, arsenic-information sources, and poor health. Concern about potential health hazards (concern) was not included as an explanatory variable because the focus was on awareness of specific health concerns. Distance to water sources was not conjectured to have an effect on perceived arsenic risks. Avoidance of exposure (switch source) was not conjectured to explain knowledge of illnesses. It may be the case that avoidance is explained by knowledge of illnesses, but knowledge of illnesses is not predicated on people's avoidance of arsenic exposure.

Avoidance of arsenic exposure (switch source) is a variable that indicates a household's decision to switch away from use of red tubewells. Switching of households from use of tubewell (switch source) was specified as a function of children, age, elderly, W13_25, W26_49, education, TW red, arsenic-information sources, concern, poor health, TW distance, and SW distance. We conjectured that sociodemographic characteristics, such as presence of children, age, presence of women, knowledge, and level of education, increased the propensity to avoid arsenic exposure. We also conjectured that concern about potential health hazards (concern) would have a positive affect on the tendency to avoid arsenic exposure. Perceived convenience, such as distance travelled to avoid arsenic exposure, of avoidance measures was also considered to have a positive affect on the tendency to avoid arsenic exposure. Prior empirical work on the avoidance of exposure to contamination showed that the presence of children, age, education, information on exposure and its consequences, subjective consideration of risk, and perceived convenience of averting activities seemed to increase the propensity to avoid exposure (10, 12–17).

All equations and analyses were done using SAS.

## RESULTS

The demographic statistics of the sample are presented in Table 2. Most respondents interviewed were female (78%), whereas the proportion of females in the overall population was 49% (18). The target respondent was the household member most knowledgeable about sources of drinking-water and water-use patterns. Since this was usually the female head of the household, the sample population had a higher proportion of women than that of the overall population of Bangladesh. The average age of the respondents was 43 years. A relatively few respondents were aged above 65 years. In contrast, only 3.4% of the overall population included persons aged over 60 years.

**Table 2.** Sample demographic statistics, random sample

Block A	
Demographic statistics	Percentage
Female	78
Age (years)	
18–35	33
35–55	47
55–65	12
>65	8
Elderly	44
Children in household	76
Education	
None	46
Up to 5^th^ grade	25
Up to 10^th^ grade	20
Up to 11^th^ grade and up	8

Almost half of the respondents were illiterate, while about one-fourth of the population had been educated only up to the fifth grade. This is roughly comparable to a literacy rate of 43.1% in the overall population.

**Fig. 2.**
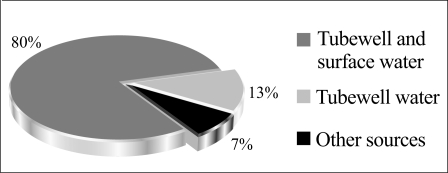
Drinking- and cooking-water sources

Eighty percent of the respondents used a combination of tubewell water and surface water for drinking and cooking (Fig. 2). Thirteen percent relied solely on tubewell water. The remaining 7% used other sources for drinking- and cooking-water.

The results of the survey revealed that approximately 70% of the respondents had exposure to arsenic problems in their households. Although this is lower than the finding of Ahmad *et al.* that 87% of respondents were aware of the arsenic problem; it is important to note that the questions asked in the two surveys were not exactly the same. Ahmad asked for awareness of arsenic (8), and here the question was whether households knew that they had an arsenic problem in their drinking-water. The survey results also showed that 64% of the respondents had switched away from use of tubewells, and 60% of these respondents had switched away from use of tubewells due to contamination with arsenic. This is also lower than the finding of Ahmad *et al*. that 69% of households in their sample area changed their drinking-water sources due to arsenic contamination (8).

Results from the first probit model indicated that the status of tubewell, arsenic-information sources, poor health, and tubewell distance were important determinants in knowledge of an arsenic problem in the household (household arsenic problem), while sociodemographic characteristics were not significant (Table 3). If the tubewell was painted red, this increased the individuals' knowledge of an arsenic problem in the household. The greater the number of sources from which the household learned about arsenic risks, the higher the individuals' knowledge of an arsenic problem in the household. People with poor health had a higher likelihood of knowing about an arsenic problem in the household. People who were using tubewells farther away also had a higher likelihood of having knowledge of an arsenic problem in the household, which might reflect the fact that people were travelling to a tubewell that was farther away to avoid exposure to arsenic in their household's water. None of the sociodemographic characteristics affected the households' knowledge of an arsenic problem.

**Table 3.** Coefficient estimates for knowledge of an arsenic problem in the household (household arsenic problem)

Variable	Coefficient estimate
INTERCEPT	0.5193
	(0.2019)†
Children	0.0652
	(0.0823)†
Age	-0.0015
	(0.0025)†
Elderly	-0.0842
	(0.0707)†
W13_25	-0.0387
	(0.0669)†
W26_49	-0.1319
	(0.0876)†
Education	0.0305
	(0.0374)†
TW red	1.3619**
	(0.0801)†
Arsenic information sources	0.0908**
	(0.0294)†
Concern	-0.0102
	(0.0702)†
Poor health	0.1493**
	(0.0257)†
TW distance	0.0011**
	(0.0002)†
SW distance	-0.0001
	(0.0002)†

**Indicates significance at 1% or lower;

†Indicates standard errors

SW=Surface water;

TW=Tubewell

Results from the second probit model explaining the knowledge of illnesses of arsenic exposure (gangrene, cancer, death, and skin lesions) are presented in Table 4. Arsenic-information sources had a significant and positive effect on knowledge of all four health consequences from arsenic exposure (gangrene, cancer, skin lesions, and death). Poor health had a significant and positive effect on knowledge of gangrene, cancer, and death. Age, presence of women aged 26–49 years, education, and arsenic-information sources—all had a significant effect on knowledge of skin lesions as a consequence of arsenic exposure. Older people tended to believe less in the probability of contracting skin lesions from arsenic exposure. Given the long latency period before skin lesions from arsenic exposure developed, an older person might perceive less risk of contracting skin lesions from arsenic exposure. The presence of women aged 26–49 years in the household increased the probability of perceiving skin lesions as a potential health hazard from arsenic exposure. Respondents with higher education and with exposure to more sources of knowledge regarding arsenic risks had a higher probability of believing that skin lesions are caused by arsenic exposure. Given that skin lesions are the most visible among the health consequences of arsenic exposure, the strength of results for knowledge of skin lesions was not surprising. The presence of children in the household had a significant and positive effect on the probability of perceiving death as a serious health hazard from arsenic exposure. Knowledge of a household arsenic problem, however, had a significant and unexpected effect on death. It decreased the probability of perceiving death as a serious health hazard from arsenic exposure.

**Table 4.** Coefficient estimates for knowledge of illnesses associated with arsenic exposure

Variable	Coefficient estimates			
	Gangrene	Cancer	Skin lesions	Death
INTERCEPT	-1.7870**	-1.4528**	1.1765**	-1.1663**
	(0.2330)†	(0.1769)†	(0.1765)†	(0.1716†
Children	-0.1046	-0.0849	-0.0570	0.1786*
	(0.0904)†	(0.0689)†	(0.0703)†	(0.0697)†
Age	-0.0014	0.0009	-0.0146**	0.0007
	(0.0029)†	(0.0022)†	(0.0021)†	(0.0021)†
Elderly	0.1011	-0.0282	-0.0139	0.0058
	(0.0778)†	(0.0591)†	(0.0607)†	(0.0575)†
W13-25	-0.0643	0.0576	0.0274	0.0025
	(0.0735)†	(0.0559)†	(0.0563)†	(0.0545)†
W26-49	0.0889	-0.0458	0.2190*	-0.0422
	(0.0996)†	(0.0764)†	(0.0748)†	(0.0721)†
Education	-0.0611	0.0564	0.1186*	0.0436
	(0.0393)†	(0.0291)†	(0.0312)†	(0.0289)†
Arsenic-informations source	0.0661*	0.0483*	0.0934*	0.0758*
	(0.0311)†	(0.0237)†	(0.0249)†	(0.0235)†
Poor health	0.1068*	0.0817*	0.0139	0.0695*
	(0.0289)†	(0.0216)†	(0.0215)†	(0.0211)†
Household arsenic problem	-0.0778	0.0197	0.0088	-0.1589*
	(0.0786)†	(0.0606)†	(0.0619)†	(0.0583)†

**Indicates significance at 1% or lower;

*Indicates significance at 5% or lower;

†Indicates standard error

Results from the third probit model (switch source) showed that the probability of avoiding arsenic exposure was influenced by education, status of tubewell, poor health, distance to tubewell, and distance to surface water (Table 5). Higher educational levels increased the probability of avoiding arsenic exposure. People who still used a red-painted tubewell were less likely to switch to an alternative source. Poor health status also decreased the probability of avoidance—it might be the case that persons with poor health were unable to walk a long distance to collect cleaner water. Distance to tubewell currently in use had a positive effect—this means that travelling a long distance to gather clean tubewell water does not deter an individual's propensity to avoid exposure. The farther away the surface water, the lower the probability of avoiding arsenic exposure. If the surface water was too far away, people likely consider switching away from using their current tubewells to be inconvenient. This may be due to the time and opportunity costs associated with the treatment of surface water and the long distance that must be traversed to get to surface water.

**Table 5.** Coefficient estimates for households switching away from use of arsenic-contaminated tubewell (switch source)

Variable	Coefficient estimate
INTERCEPT	-0.0239
	(0.1883)†
Children	0.0655
	(0.0773)†
Age	-0.0025
	(0.0024)†
Elderly	-0.0192
	(0.0654)†
W13_25	-0.0577
	(0.0616)†
W26_49	-0.0372
	(0.0798)†
Education	0.0710*
	(0.0351)†
TW red	-0.8938**
	(0.0630)†
Arsenic-information sources	-0.0141
	(0.0270)†
Concern	-0.0034
	(0.0644)†
Poor health	-0.0455*
	(0.0233)†
TW distance	0.0045**
	(0.0003)†
SW distance	-0.0010*
	(0.0004)†

**Indicates significance at 1% or lower;

*Indicates significance at 5% or lower;

†Indicates standard error

## DISCUSSION

Awareness campaigns in Matlab (word of mouth or education programmes) increased knowledge of an arsenic problem in the household. This result confirms the hypothesis of Jalan that household awareness can be raised through education imparted directly and indirectly by public-awareness campaigns (1). In addition, the results of this analysis suggest that the sociodemographic profile of a household does not affect the propensity of a household to be influenced by awareness campaigns. According to this study, people's education, age, or gender had no effect on knowledge of an arsenic problem in the household. Risk-communication efforts were effective regardless of the specific sociodemographic characteristics investigated in this research.

The likelihood of perceiving serious health hazards from arsenic exposure was also strongly affected by efforts (word of mouth or awareness campaigns) to raise public awareness. We found a variation in how sociodemographic characteristics affected the knowledge of specific illnesses caused by arsenic exposure. The likelihood of perceiving gangrene and cancer was not influenced by sociodemographic characteristics. The health consequence most strongly influenced by sociodemographic characteristics was skin lesions. A sociodemographic parameter that affected the likelihood of perceiving death due to arsenic exposure was the presence of children in the household. This supports the findings of Abdallah *et al.* who found that households were more likely to avoid exposure if they knew about the contamination, if they perceive that the risks of cancer would increase, and if they have children in the household (12). The findings in our study showed that exposure to more sources of knowledge raised subjective considerations of risk. In addition, the likelihood of perceiving death as a serious health hazard from arsenic exposure was higher for households that had one or more child(ren) in the family. This supports the conjecture that households perceive a higher health risk from arsenic exposure if there are children in the household.

The likelihood of avoiding exposure to arsenic in drinking-water was largely unaffected by sociodemographic characteristics (except for education). In addition, information on exposure from various sources did not affect the likelihood of avoiding exposure. This does not support previous findings that show that information on exposure and subjective consideration of risk increases the tendency to take defensive action (13–17). In particular, the conjecture that households are more likely to take actions to avert arsenic exposure if there are children to consider is not supported. Although the households perceived a higher health risk from arsenic exposure if there were children in the household, avoidance measures as defined in this study are not likely to be adopted. The factors that affected the likelihood of avoiding exposure were poor health and distance to alternative sources for water. The results showed that people were willing to walk a long distance to avoid exposure if the source for arsenic-free water was a tubewell. If the source for arsenic-free water was surface water, however, people were less likely to walk a long distance to take avoidance measures. Consumption of surface water requires further avoidance measures (such as boiling) to avoid contamination by pathogens. People may not consider the trade-off of health risks for consuming pathogen-contaminated surface water to be worth the perceived benefit of avoiding arsenic exposure. Collecting surface water as an avoidance measure may simply be too inconvenient. Persons with poor health may also find it inconvenient to travel a long distance to collect cleaner water. This study has shown that the major factors affecting the propensity to avoid arsenic exposure are convenience and education. The analysis concurs with Dasgupta and McConnell and Rosado's who found that higher-educated individuals had a higher likelihood of averting exposure (19, 20) and also with Laughland *et al.* who found that averting actions were positively related to the perception of convenience of averting practices (16, 19, 20).

Convenience is a major factor, then, to be taken into consideration for public health-mitigation policy and public-health mitigation measures. According to this analysis, awareness campaigns had the desired effect in terms of informing the public. They did not, however, go far enough to affect the behaviour or actions that individuals undertake to protect themselves from arsenic exposure. Given competing needs, it cannot be assumed that if people are better informed on their exposures to arsenic risk factors, they will necessarily act to change their exposure to arsenic risks. Although the results of the study are specific to Matlab, Bangladesh, the general findings are of relevance to other rural areas around the developing world.

## ACKNOWLEDGEMENTS

The authors thank Rowen Aziz for her invaluable technical assistance. This work was supported and funded by the Maine Agricultural and Forest Experiment Station (MAFES), University of Maine, USA and ICDDR,B.
